# Optimizing the Use of Basil as a Functional Plant for the Biological Control of Aphids by *Chrysopa pallens* (Neuroptera: Chrysopidae) in Greenhouses

**DOI:** 10.3390/insects13060552

**Published:** 2022-06-16

**Authors:** Yan Fang, Shu Li, Qingxuan Xu, Jie Wang, Yajie Yang, Yingying Mi, Zhenyu Jin, Nicolas Desneux, Su Wang

**Affiliations:** 1 Institute of Plant Protection, Beijing Academy of Agriculture and Forestry Sciences, Beijing 100097, China; fangyan1998@126.com (Y.F.); lishu@ipepbaafs.cn (S.L.); xuqingxuan@ipepbaafs.cn (Q.X.); wj_insect@126.com (J.W.); duoduoyang9755@163.com (Y.Y.); 15572177930@163.com (Y.M.); 2 Forewarning and Management of Agricultural and Forestry Pest, Hubei Engineering Technology Center & College of Agriculture, Yangtze University, Jingzhou 434025, China; 3 College of Agronomy, Sichuan Agricultural University, Chengdu 611130, China; 4 Institut Sophia Agrobiotech, Université Côte d’Azur, INRAE, CNRS, UMR ISA, 06000 Nice, France; nicolas.desneux@inrae.fr

**Keywords:** *Chrysopa pallens*, *Myzus persicae*, basil, conservation biological control, intercropping, spatial arrangement, greenhouse

## Abstract

**Simple Summary:**

Functional plants can be deployed at the field, farm, and landscape scale, where they are beneficial to natural enemies, thus contributing to improved pest control. To explore how non-crop plants can augment the biological control of pests, this study aimed to assess how basil (*Ocimum basilicum* L.) (Lamiales: Lamiaceae), as a functional plant, affected the lacewing *Chrysopa pallens* (Rambur) (Neuroptera: Chrysopidae) in the laboratory and greenhouse. The results showed that in the presence of the target prey (peach aphid; *Myzus persicae* (Sulzer)), both the vegetative and flowering stages of basil enhanced *C. pallens* (early-age) fecundity and longevity as compared to a control treatment in the laboratory. Similarly, lacewing colonization patterns were modulated by the basil planting density and spatial arrangement in the greenhouse. Under high density intercrop basil arrangements, *C. pallens* colonization rates were the highest, the populations persisted longer in the crop, and the aphid numbers declined more rapidly. This work showed how basil enhanced the fitness attributes of a generalist predatory lacewing and benefitted aphid biological control in a short time. It can inform the development of economically sound management strategies to attain pest control with minimum inputs.

**Abstract:**

Effective biological control agents that can provide sustainable pest control need to be researched in further detail; functional plants (or non-crop insectary plants), in particular, are garnering increased research interest. Much remains to be learned as to how non-crop plants can augment biological control in greenhouse systems. In this study, we combined laboratory and greenhouse assays to assess the extent to which basil (*Ocimum basilicum* L.) (Lamiales: Lamiaceae) affected the biological control of aphids by the predatory lacewing *Chrysopa pallens* (Rambur) (Neuroptera: Chrysopidae). In the presence of the target prey (peach aphid; *Myzus persicae* (Sulzer)), both the vegetative and flowering stages of basil enhanced *C. pallens* longevity and (early-age) fecundity as compared to a control treatment. When basil plants were established near aphid infested eggplants (*Solanum melongena* L.), the *C. pallens* colonization rate improved by 72–92% in the short-term. Lacewing colonization patterns were modulated by the basil planting density and spatial arrangement (i.e., perimeter planting vs. intercropping). Under high density intercrop arrangements, *C. pallens* colonization rates were highest, its populations persisted longer in the crop, and the aphid numbers declined more rapidly. Our work shows how basil enhanced the key fitness attributes of a generalist predatory lacewing and benefitted aphid biological control in a greenhouse setting.

## 1. Introduction

Biological control constitutes a viable, environmentally friendly, and economically sound alternative to pesticide-based measures for agricultural pest management [1] without the side effects linked to these pesticides [2,3]. However, most attempts to use biological control agents have been unsuccessful, largely because of the local environmental adaptation of the agent [4]. More and more research has focused on how augmentative applications of biological control agents (BCAs) and/or their in-field conservation can provide sustainable pest control and reduce the risk of failure [5,6,7,8]. The insectary and/or functional plants provide the resident or artificially released BCAs with alternative prey/host items, nutritional resources, and refuge [9,10,11], thus benefiting the BCAs’ field colonization dynamics [12,13]. To fully tap these biological control benefits, the selection of functional plant species needs to be scientifically informed and based upon a sound understanding of ecological mechanisms [13,14,15]. Though their use is relatively well studied for arable crops in the framework of conservation biological control [16,17,18], their potential use for protected crops is less studied. Some attention has been paid to the deployment of insectary plants at the periphery of greenhouses [19,20]. Similarly, ways have been investigated to use banker plants infested with non-pest alternative prey to aid BCA establishment [21,22]. Indeed, plants’ spatial arrangement can affect volatile release patterns and modulate natural enemy foraging dynamics [23,24,25]. However, the efforts of functional plant arrangements on biological pest control are still unknown.

While greenhouse vegetables are a pivotal component of the local food system in many countries, they also provide suitable environments for pest development and proliferation. The green peach aphid *Myzus persicae* (Sulzer) (Hemiptera: Aphididae) is one of the most serious pests of various vegetables in greenhouse environments. It causes considerable damage to crops and transmits various plant viruses, resulting in significant economic losses. More effective BCA are particularly pressing in greenhouse settings where high value crops, e.g., flowers or vegetables, are grown under space constraints [19]. As greenhouse grown vegetables are high value commodities, the relative merits of functional plants need to be closely scrutinized. By providing science based advice on planting densities or spatial arrangements, one can optimize their contribution to pest management [8]. Therefore, we need more precise and accurate application of functional plants to enable them to play the role of regulating natural enemies, improving the efficiency of colonization and damage control within a short time.

One of the most common BCAs in China is the lacewing, *Chrysopa pallens* (Rambur) (Neuroptera: Chrysopidae). This generalist predator, which is widely deployed for biological control in agriculture and forestry [26], engages in predation during both the adult and larval stages [27]. Given the dispersal capabilities of *C. pallens*, a timely colonization of the focal crop is key to ensure successful biological control [28]. This can potentially be attained through the deployment of functional plants, e.g., insectary or banker plants. Previous studies have evaluated the reactive preference of lacewing *C. pallens* to aromatic plant basil (*Ocimum basilicum* L.) [29], which is widely distributed worldwide and routinely grown in greenhouses. Not only do the essential oils from *O. basilicum* exhibit a repellent effect on different pests [30], but the plant also secretes copious amounts of energy rich nectar [31]. However, no previous studies have evaluated how establishment of basil plants in greenhouse settings could ensure the timely effective action of resident or released natural enemies to secure economically sound biological control.

Therefore, we hypothesized that (1) the presence of basil, as a functional plant, could enhance the lacewing’s longevity and fecundity, and (2) the basil planting density and spatial arrangement in the greenhouse would affect the control effect of *C. pallens*. We conducted laboratory trials to assess whether the vegetative and flowering stages of basil affected different *C. pallens* fitness parameters (i.e., fecundity, longevity of female), and paired greenhouse assays to investigate the extent to which basil contributes to lacewing developmental fitness and the ensuing biological control. 

## 2. Materials and Methods

### 2.1. Insects and Plants

A laboratory colony of *C. pallens* was established at the Institute of Plant Protection (IPP), Beijing Academy of Agricultural and Forestry Sciences (BAAFS), in Beijing, China. The colony was founded in June 2019 by live lacewing individuals collected from a maize field at the Noah Organic Farm (116°59′ E, 40°6′ N) in Pinggu County, Beijing. Rearing protocols were adapted from Wang et al. [26]. In brief, adults and immatures of lacewings were kept at a density of 30–40 pairs in 60 cm × 60 cm × 60 cm aluminum cages with plastic 80-mesh screen walls. They were fed with sufficient bean aphid *Megoura japonica* (Matsumura) on young broad bean (*Vicia faba* L.) plantlets. Plantlets were refreshed twice a week. Every day, adult rearing cages were visually inspected and *C. pallens* eggs were gently collected from bean plants and transferred into another cage for egg eclosion, juvenile development, and pupation. Newly emerged adults were selected for experimental assays. 

Apterous aphids of the key pest species, *M. persicae* [32], were reared on sprouts of radish (*Raphanus sativus* L.) in a climate controlled chamber at 25 ± 2 °C, 60 ± 10% RH, 16L:8Dh photoperiod, and 1000 lux. Each radish seedling had about 25 aphids. Eight-day old aphids were used in the greenhouse experiments. 

Basil (*O. basilicum*) seeds were purchased from the Beijing Vegetable Research Center, BAAFS. The basil was seeded in plastic trays, on a substrate composed of vermiculite, perlite, and peat soil (1:1:4 ratio). Once the seedlings obtained 3–4 true leaves, they were individually transplanted into plastic pots (height 20 cm, diameter 13 cm). All plants were kept at 25 ± 2 °C, 60 ± 10% RH, and a L16: D8 h photoperiod and were watered twice per week. In laboratory trials, both the vegetative and flowering stages of *O. basilicum* were used. Thirty day old basil plants (i.e., 30–35 cm high; 5–7 expanded leaves) were chosen as the ‘vegetative stage’, while 60-day old plants with open flowers were termed the ‘flowering stage’.

### 2.2. Laboratory Trials

A laboratory trial was carried out to assess the extent to which the vegetative and flowering stages of basil affected different *C. pallens* fitness parameters. Specifically, the following treatments were established: (1) a pot of basil in the vegetative stage + a pot of broad bean seedlings with *M. japonica*, (2) a pot of basil in the flowering stage + a pot of broad bean seedlings with *M. japonica*, and (3) a pot of broad bean seedlings free of aphids + a pot of broad bean seedlings with *M. japonica*. Both plants were introduced into a 35 cm × 35 cm × 55 cm cage, which was made of 80-mesh plastic net and aluminum alloy frames. One pair (1 male + 1 female) of newly emerged *C. pallens* adults were starved for 24 h and then released into the cage. The cages were kept in a climate-controlled chamber at 25 ± 2 °C, 60 ± 10% RH, and L16: D8 h. Experiments were run until the death of the *C. pallens* female, and the pots of broad bean seedlings with 800~1000 *M. japonica* were replaced every day. On a daily basis, female survival and the number of deposited eggs was assessed. The total (lifetime) fecundity and the fecundity over the initial five days of lacewing females was, thus, determined. Each treatment was established with 15 replicates (*n* = 15).

### 2.3. Greenhouse Assays

A greenhouse assay was carried out to assess whether the planting density and spatial arrangement of basil plants affected lacewing colonization rates, population persistence, and *M. persicae* control. Assays were carried out in 18 isolated mesh tunnels at Noah Organic Farm. Seeds of eggplant (*Solanum melongena* L.) variety ‘Jingqie No.13’ were obtained from the Beijing Seed Sci-Tech Co. Ltd. (Beijing, China) and sown in plastic trays on 3 April 2019. On 15 May, the eggplant broad was established in two 8-plant rows (inter-row space 80 cm). Five eggplant broads were in each mesh tunnel. The spacing between the mesh tunnels was 8 m. Plants were subject to fertigation, which enabled optimum plant development and circumvented abiotic stress. Pesticide applications were strictly avoided.

**Figure 1:**
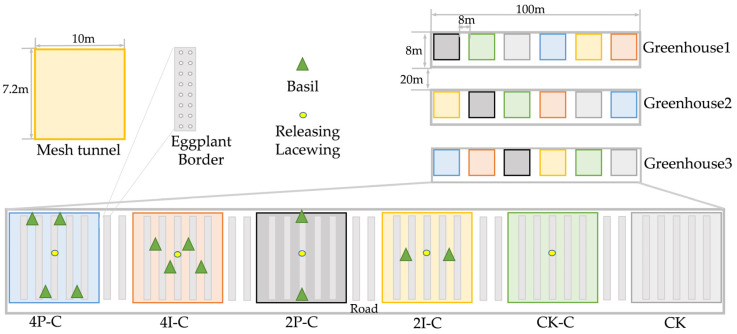
Illustration of the experimental design of the enhancement effect of basil *O. basilicum* on the lacewing *C. pallens* in colonization and pest suppression in a greenhouse. Each greenhouse was 100 m × 8 m and divided into 6 10 m × 7.2 m mesh tunnels. The spacing between the mesh tunnels was 5 m. The six colors of the mesh tunnels indicate the different treatments: 4P-C, four basil plants placed around the perimeter of the eggplant, with five pair of lacewing adults released; 4I-C, four basil plants intercropped with the eggplant, with lacewing adults released; (3) 2P-C, two basil plants placed around the perimeter of the eggplant, with lacewing adults released; (4) 2I-C, two basil plants intercropped with the eggplant, with lacewing adults released; (5) CK-C, no basil plants, with lacewing adults released (control-C); and (6) CK, no basil plants and no lacewing (control).

Treatments were designed based on the spatial arrangement (i.e., perimeter or intercropping) and density (four or two plants) of the basil plants, and the control treatments CK-C and CK in the presence and absence of *C. pallens*, respectively (Figure 1). Six treatments were compared: (1) 4P-C, four basil plants placed around the perimeter of the eggplant, with five pair of lacewing *C. pallens* adults released; (2) 4I-C, four basil plants intercropped with the eggplant, with five pair of lacewing *C. pallens* adults released; (3) 2P-C, two basil plants placed around the perimeter of the eggplant, with five pair of lacewing *C. pallens* adults released; (4) 2I-C, two basil plants intercropped with the eggplant, with five pair of lacewing *C. pallens* adults released; (5) CK-C, no basil plants, with five pair of lacewing *C. pallens* adults released (control-C); (6) CK, no basil plants and no lacewing (control). One single treatment was established within each mesh tunnel (10 m (L) × 7.2 m (L) × 2 m (H)), which was replicated three times (*n* = 3) under a random block design in each of three selected greenhouses (100 m × 8 m). At the onset of the experiment, 100 aphids *M. persicae* were gently brushed onto the eggplants per treatment group and allowed to establish. On the third day, five pair of newly emerged *C. pallens* adults were released into each tunnel (except for the CK treatment), and the number of aphids in each treatment was investigated. Next, the number of aphids and lacewing on eggplant plants was visually recorded every 3 days. A Z-sampling method was adopted, and 15 points were surveyed for each treatment at 8:00–10:00. At each point, two eggplant plants were selected, and two leaves were randomly chosen from the upper, middle, and lower layer of the plant. On each leaf, the number of lacewing and aphid individuals was recorded. Insect surveys were thus conducted between 25 June and 10 July 2019.

### 2.4. Statistical Analysis

In laboratory assays, the effect of the basil phenological stage on *C. pallens* fecundity was determined using one-way analysis of variance (One-Way ANOVA), followed by Fisher’s LSD test (*p* < 0.05). The effect of the different experimental treatments on the longevity of female and male lacewing adults was analyzed by Kaplan–Meier estimators (log-rank method) followed by multiple comparisons of survival curves with the Fisher’s LSD test. In the greenhouse assays, the percentage of aphid control, the relative effect of establishing the basil functional plants, and the lacewing colonization rate were computed as follows:

Total control effect (%) = [(aphid abundance in CK − aphid abundance in the treatment)/(aphid abundance in CK)] × 100;

Functional plant effect (%) = [(aphid abundance in CK-C − aphid abundance in the treatment)/(aphid abundance in CK-C)] × 100;

Lacewing colonization rate (%) = (lacewings recorded on plants/lacewings released) × 100.

Analysis of variance (ANOVA) was used to assess the colonization rate of *C. pallens*, the total control effect, and the functional plant effect between the five treatments (4I-C, 4P-C, 2I-C, 2P-C, and CK-C). Fisher’s LSD was used to separate the treatment differences in terms of means at the 0.05 level. Then, to determine effect of basil density, spatial arrangement, and basil density × spatial arrangement, the aphid and lacewing abundance in four treatments (4I-C, 4P-C, 2I-C, and 2P-C) were also tested using ANOVA. Data were checked for normality and homoscedasticity as appropriate and, if needed, were log-transformed. All statistical analyses were carried out with SPSS Statistics 23.0 statistical software (IBM). The survival rates under the different experimental treatments were plotted using Sigma plot 10.0, and the other diagrams were drawn using Origin 2018.

## 3. Results 

### 3.1. Laboratory Trials

**Figure 2:**
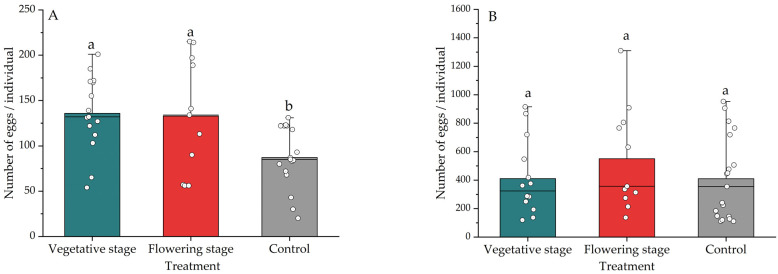
Effect of the *Ocimum basilicum* phenological stage on *Chrysopa pallens* fecundity. (**A**) shows the *C. pallens* egg deposition rates during the first 5 days, while (**B**) indicates the total (lifetime) egg deposition per female. Different letters indicate significant differences between treatments (ANOVA, Fisher’s LSD test for mean separation, *p* < 0.05).

**Figure 3:**
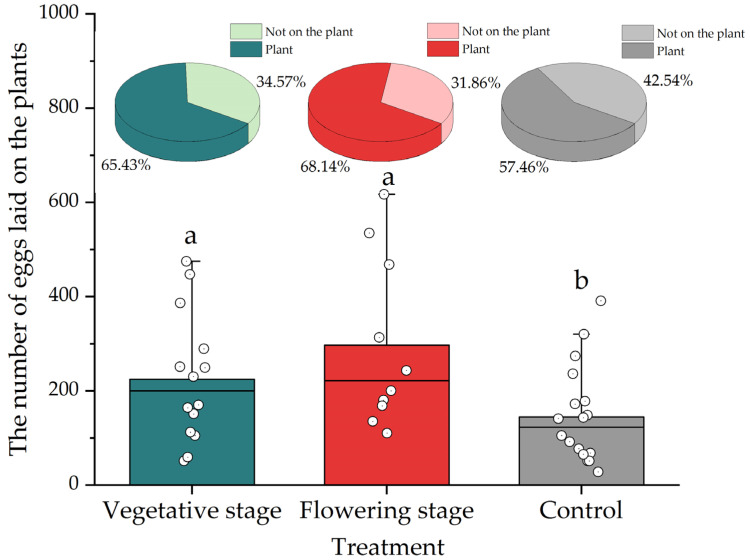
Effect of the *Ocimum basilicum* phenological stage on the number of *Chrysopa pallens* eggs that were deposited on plants, e.g., as compared to cage walls or pots. Results are shown for *O. basilicum* vegetative and flowering stages, as compared to the control treatment. Different letters indicate statistically significant differences between treatments (ANOVA, Fisher’s LSD test for mean separation, *p* < 0.05).

**Figure 4:**
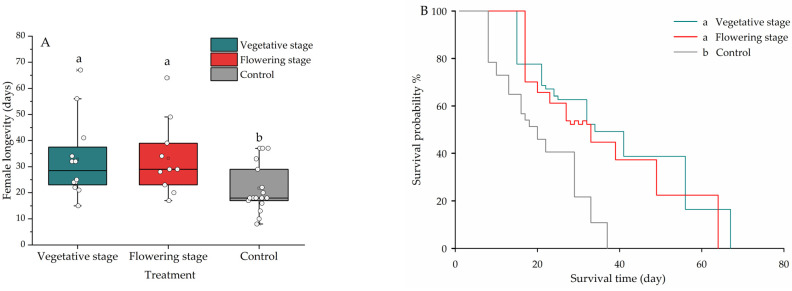
Effect of *Ocimum basilicum* phenological stage on the (**A**) longevity and (**B**) survival of female *Chrysopa pallens* adults. The results are shown for vegetative and flowering *O. basilicum*, as compared to the control treatment. Kaplan–Meier curves are drawn to depict the *C. pallens* survival dynamics under different treatments. Different letters indicate statistically significant differences among treatments (ANOVA, Fisher’s LSD test for mean separation, *p* < 0.05).

In the presence of aphid prey, the vegetative or flowering basil did affect lacewing fecundity during the initial five days (*F*_2,49_ = 4.015, *p* = 0.025) (Figure 2A). However, the basil did not affect total (i.e., lifetime) lacewing fecundity (*F*_2,44_ = 0.884, *p* = 0.419) (Figure 2B). In the presence of basil, a higher number of eggs were also deposited on basil, e.g., as compared to the cage wall (*F*_2_,_40_ = 3.536, *p* = 0.039) (Figure 3). More specifically, lacewings deposited 65.4%–68.1% of eggs on the plants in the presence of basil, as compared to 57.5% in the absence of basil (Figure 3). The treatments equally affected *C. pallens* female longevity (*F*_2,36_ = 3.762, *p* = 0.033): in the presence of (vegetative and flowering) basil plants, longevity increased by 32.36% (Figure 4A), and the survival probability increased by 77% (Figure 4B). While female adults survived for 38 days in the control treatment, they lived for 67 and 64 days in the presence of vegetative and flowering basil plants, respectively.

### 3.2. Greenhouse Assays

**Figure 5:**
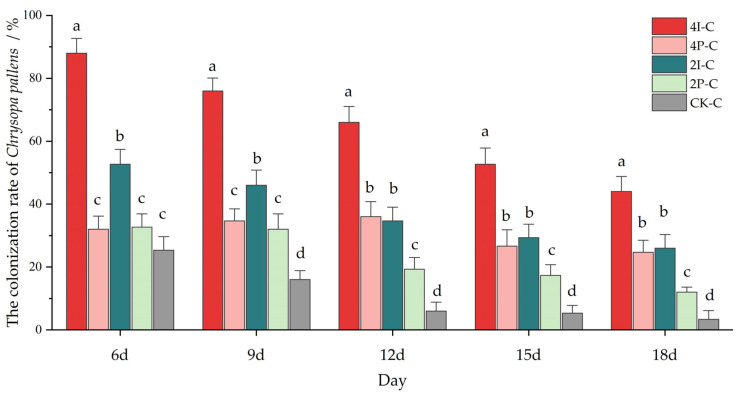
The colonization rate of *Chrysopa pallens* in greenhouse-grown eggplant crops with different spatial arrangements and densities of *Ocimum basilicum*. The colonization rate refers to the retention rate of the lacewings that were initially released at the start date. The bar charts show mean ±SE values. Different letters above a given bar reveal statistically significant differences between treatments (*p* < 0.05).

In the presence of basil plants, the lacewings attained higher colonization rates in the greenhouse tunnels (Figure 5). Six days after the onset of the experiment, the lacewing abundance declined to 75% in the CK-C. Over time, the spatial arrangement and planting densities of the basil affected the *C. pallens* colonization patterns (6d: *F_4,70_* = 74.126, *p* < 0.001; 9d: *F_4,70_* = 64.741, *p* < 0.001; 12d: *F_4,70_* = 63.363, *p* < 0.001; 15d: *F_4,70_* = 38.328, *p* < 0.001; 18d: *F_4,70_* = 40.027, *p* < 0.001) (Figure 5). Meanwhile, *C. pallens* consistently attained the highest colonization rate in the intercropped basil at high densities (i.e., treatment 4I-C). The spatial arrangements and densities of *O. basilicum* significantly affected the numbers of lacewings *C. pallens*; the interaction also had a significant effect in four treatments (4I-C, 4P-C, 2I-C, and 2P-C) for 15 days (*p* < 0.05) (Table 1).

**Figure 6:**
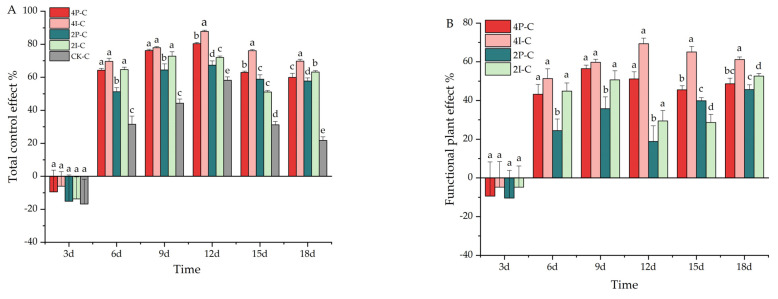
The percentage of aphid control and the relative effect of establishing basil functional plants. (**A**) Total control effect; (**B**) functional plant effect. The bar charts show mean ± SE values. Different letters above a given bar reveal statistically significant differences between treatments (*p* < 0.05).

The basil spatial arrangement and planting density both affected the *M. persicae* numbers, though the interaction term was nonsignificant at 9d, 12d, and 18d (Table 1). On the third day after lacewing release, there was no difference in the total control effect (*F_4,70_* = 0.107; *p* = 0.980) or functional plant effect (*F_3,56_* = 0.044; *p* = 0.987) (Figure 6). At later times, however, the total control effect (Figure 6A) (6d: *F_4,70_* = 31.595, *p* < 0.001; 9d: *F_4,70_* = 33.560, *p* < 0.001; 12d: *F_4,70_* = 49.808, *p* < 0.001; 15d: *F_4,70_* = 72.108, *p* < 0.001; 18d: *F_4,70_* = 110.074, *p* = 0.001) and the functional plant effect (Figure 6B) (6d: *F_3,56_* = 5.171, *p* = 0.003; 9d: *F_3,56_* = 6.858, *p* < 0.001; 12d: *F_3,56_* = 17.366, *p* < 0.001; 15d: *F_3,56_* = 28.466, *p* < 0.001; 18d: *F_3,56_* = 9.980, *p* < 0.001) showed significant differences.

## 4. Discussion

Aromatic plants emit volatile cues and can provide energy rich floral nectar for multiple predator and parasitoid species [33]; their establishment in standing horticulture crops can potentially bolster biological pest control. The present study reveals how both the vegetative and flowering stages of basil raised different fitness parameters (i.e., longevity and fecundity) of a generalist predator, i.e., the lacewing *Chrysopa pallens*. By establishing the basil plants at a high density (i.e., 0.4 per m^2^) in tunnel greenhouses, the *C. pallens* colonization rates were strongly enhanced, and the population persistence was improved. Augmentative releases of *C. pallens* under basil high density intercrop arrangements yielded high levels of control over relatively short time spans. Our work also revealed how the time, spatial distribution, and density of functional plants in a closed greenhouse system jointly determined biological control outcomes.

Functional plant research has often focused on plants’ blooming period [34,35]. Indeed, floral nectar and pollen positively affect the development, foraging behavior, and biological control impact of different natural enemies [10,36,37,38], including hoverflies, ladybeetles, mirid bugs, and lacewings [36,39,40]. These beneficial impacts often become more apparent in the presence of target prey. For example, when access to prey is allowed, flowering aromatic plants bolster survival and raise the early-age fecundity of predatory ladybeetles [40]. For many omnivorous natural enemies, mixed diets of animal prey and plant-based resources deliver major fitness benefits [41]. However, even for lacewing omnivores, the impacts of plant based resources were inconsistent and were modulated by prey densities [42]. Our study highlights how flowering basil plants and possibly its pollen or nectar raised lacewing fitness but also shows that the vegetative stages of this plant increased the fecundity and survival rates of *C. pallens*. In the absence of extra floral nectary [43], *C. pallens* potentially gains subtle fitness benefits by feeding on other plant tissues in a similar way to predaceous Heteroptera [44]. These findings are particularly important, as the blooming period of basil and many functional plants only cover a short part of the annual life cycle. Irrespective of the underlying mechanics, this phenomenon is unreported in the literature and merits further in depth investigation. Therefore, our study focused on the different growth periods of plants, taking into consideration the selection of functional plants, which are effective for natural enemies at suitable stages. In the process of crop production, we can cultivate functional plants in advance and select their best application period, including the vegetative and flowering stages, which can be applied during the whole crop production cycle. By selecting functional plants that also benefit natural enemies during their vegetative stages or that repel target pests [45], one can extend their contribution to non-chemical pest management. Overall, these synergisms should be actively pursued in order to maximize the potential of functional plants in biological control. 

Natural enemy foraging patterns are shaped by a range of factors, including flowering plants’ color, odor, size, architecture, the timing of nectar production, and flower corolla aperture and depth [36,46,47]. Omnivorous lacewings are attracted to a range of plant species, which they use for reproduction, feeding, or resting [48]. Their orientation behavior and plant preference are shaped by the odor blends [49] and UV patterns of flowers [50]. Lacewings also exhibit associative learning and, thus, relate food or prey availability to certain volatile signals [51]. As both vegetative and flowering basil plants stimulated lacewing landing and oviposition on nearby plants [52], constitutively produced volatile cues are possibly at play. Followup research is merited to identify these key functional traits of basil and possibly other aromatic plants, assess their relevance to biological control, and draw upon their potential when devising diversification schemes. 

Though adult oviposition patterns determine biological control efficacy [53,54], little is known about these behavioral processes for predatory lacewings. Lacewings have been observed to lay eggs near certain plant organs (e.g., corn ears and leaf edges) or in prey-infested areas [55], but these patterns are not consistent across crop x pest systems [56]. Our work revealed how, under greenhouse settings, *C. pallens* deposited eggs on various plant and non-plant structures (e.g., mesh net and planting trays). Yet, in the presence of basil plants, egg deposition on plants was augmented by 55%. Given its importance to biological control, the underlying functional trait of basil awaits empirical characterization.

Crop diversification measures (e.g., the addition of basil to an eggplant crop) can benefit resident natural enemies by providing alternative hosts and prey items, nutritional resources, or suitable microclimatic conditions [9,57,58]. Yet, natural enemy responses to diversification tactics have not been consistently translated into heightened pest suppression [59]. The effects of plant diversification are mediated by the spatial arrangements and cropping patterns [60]. In open field systems, functional plants have been examined under intercrop, perimeter, and rotation arrangements [25,61], and habitat management schemes such as conservation strips have been investigated [60,62]. Conversely, the cropping environment in greenhouse settings is relatively simple and does not appear to lend itself to manipulation over extended spatiotemporal scales. Our work, however, shows that this type of research is equally warranted in greenhouse settings, as the spatial configuration of basil companion plants dictated different levels of natural enemy colonization and pest suppression. Similarly, basil planting density mediated the biological control. Hence, specific spatial configurations with basil or other aromatic plants benefit biological control in greenhouse crop x pest systems; however, they clearly require further investigation. Future work can examine how different functional plant species can be used for a set of natural enemies or target pests. 

In open ecosystems, biodiversity and trophic interactions contribute to ecological stability and underpin biological control. However, in closed systems such as greenhouses, habitats are simplified, food chains are comparatively short, and ecological resilience is exceptionally weak. In the absence of plant diversity, pest populations rapidly proliferate and often require corrective action. Biological control in greenhouse systems can be improved by simultaneously raising the short-term colonization rates of inoculatively released natural enemies and by actively conserving their populations. Until now, most research has focused on long-term extensive control, but the study of short-term pest population control has received little consideration. Therefore, we examined the change in the pest population density in the short term, which is helpful to master the precise management of functional plants. Meanwhile, our work showed how basil (*O. basilicum*) readily bolstered the efficacy of the biological control over the short term, by offering nutritional resources and foraging cues to resident predators. Though the underlying mechanisms await further clarification, the phenological stage, density, and spatial arrangement of basil plants in an eggplant crop modulated lacewing fitness, colonization dynamics, and aphid pest control. Future work can examine how functional plants can contribute to the establishment and maintenance of viable natural enemy populations in greenhouse settings. Our work not only points toward lucrative opportunities to advance sustainable pest management in Chinese horticulture but also strengthens the evidence base to deploy functional plants in farming systems globally.

## Figures and Tables

**Table 1:** ANOVA of the effects of the density, spatiality, and their interactions on the abundance of lacewings *Chrysopa pallens* and aphids *Myzus persicae* in different spatial arrangements and densities of *Ocimum basilicum.*

Day	Source	Lacewings			Aphids		
		*F*	df	*p*-Value	*F*	df	*p*-Value
3 d	Spatiality	162.45	1	<0.001	33.9	1	<0.001
	Density	33.8	1	<0.001	30.1	1	<0.001
	Spatiality × Density	36.45	1	<0.001	6.37	1	0.014
9 d	Spatiality	87.678	1	<0.001	5.33	1	0.025
	Density	30.558	1	<0.001	14.4	1	<0.001
	Spatiality × Density	21.395	1	<0.001	2.39	1	0.128
12 d	Spatiality	56.886	1	<0.001	14.1	1	<0.001
	Density	63.775	1	<0.001	80.8	1	<0.001
	Spatiality × Density	5.954	1	0.018	0.56	1	0.457
15 d	Spatiality	38.811	1	<0.001	2.9	1	0.094
	Density	28.681	1	<0.001	74.9	1	<0.001
	Spatiality × Density	5.268	1	0.025	38.2	1	<0.001
18 d	Spatiality	42.169	1	<0.001	27	1	<0.001
	Density	35.692	1	<0.001	9.32	1	0.003
	Spatiality × Density	1.08	1	0.303	2.32	1	0.133

Notes: The effect of aphid and lacewing abundance were in basil spatial arrangement (perimeter or intercropping), density (four or two plants), and their interactions (density × spatial) arrangement.

## Data Availability

The datasets generated during the study are available from the corresponding author on reasonable request.
